# From Plaque to Perfusion: A Narrative Review of Multimodality Imaging in Acute Coronary Syndromes

**DOI:** 10.3390/jcm15082905

**Published:** 2026-04-11

**Authors:** Ahmed Shahin, Salaheldin Agamy, Sheref Zaghloul, Ranin ElShafey, Maha Molda, Zahid Khan, Luciano Candilio

**Affiliations:** 1 Cardiology Department, Hampshire Hospitals NHS Foundation Trust, Basingstoke RG24 9NA, UK; 2 Cardiology Department, Heartlands Hospital, University Hospitals Birmingham NHS Foundation Trust, Birmingham B9 5SS, UK; 3 Cardiology Department, Royal Berkshire Hospital, Reading RG1 5AN, UK; 4 Cardiology Department, Tanta University Hospitals, Tanta 31527, Egypt; 5 Radiology Department, East Cheshire NHS Foundation Trust, Macclesfield SK10 3BL, UK; 6 Townsville University Hospital, Townsville, QLD 4814, Australia; drzahid1983@yahoo.com; 7 Department of Medicine and Dentistry, James Cook University, Townsville, QLD 4814, Australia; 8 William Harvey Research Institute, Queen Mary University, London E1 4NS, UK; 9 Royal Free London NHS Foundation Trust, London NW3 2QG, UK

**Keywords:** acute coronary syndromes, artificial intelligence, coronary computed tomography angiography, intravascular imaging, myocardial infarction with non-obstructive coronary arteries (MINOCA), optical coherence tomography, percutaneous coronary intervention, plaque phenotyping, precision medicine

## Abstract

**Background**: This narrative review introduces the “From Plaque to Perfusion” framework, a clinically pragmatic approach that maps multimodality imaging technologies to critical decision points in the acute coronary syndrome (ACS) patient journey. By integrating non-invasive assessment, invasive procedural guidance, and post-event tissue characterisation, this framework provides a structured pathway for deep phenotyping of ACS. Artificial intelligence (AI) is highlighted as an essential enabling layer that enhances diagnostic precision, automates quantification, and supports scalable, data-driven care. Contemporary ACS management pathways, while effective, often leave residual clinical uncertainty. The diagnostic objective has evolved beyond confirming myocardial injury to comprehensively phenotyping the entire ACS cascade: defining the plaque substrate, identifying the culprit mechanism, and quantifying the myocardial consequence. This requires a systematic integration of advanced imaging modalities. **Methods**: This narrative review is based on a comprehensive literature search of major medical databases (PubMed/MEDLINE, Scopus, Embase, Google Scholar) for high-level evidence, including randomized controlled trials, meta-analyses, and international expert consensus documents published between January 2010 and February 2026. **Results**: The “From Plaque to Perfusion” framework consists of three core stages. First, non-invasive assessment with coronary computed tomography angiography (CCTA), fractional flow reserve (FFR-CT), and PET-CT defines plaque substrate and vascular inflammation. Second, invasive precision in the catheterization laboratory, guided by optical coherence tomography (OCT) and intravascular ultrasound (IVUS), resolves the culprit mechanism and optimizes percutaneous coronary intervention (PCI). Third, post-event tissue characterization with cardiac magnetic resonance (CMR) quantifies myocardial injury and refines prognosis. AI-driven platforms are shown to enhance each stage by automating analysis, standardizing interpretation, and providing actionable metrics for clinical decisions, including complex scenarios like Myocardial Infarction with Non-Obstructive Coronary Arteries (MINOCA). **Conclusions**: The “From Plaque to Perfusion” framework, enabled by AI, reframes ACS imaging as an integrated, mechanism-driven pathway. This approach moves beyond isolated test interpretation toward a scalable model of precision, phenotype-led care that promises to improve diagnostic certainty and personalize patient management.

## 1. Introduction: The Need for Deep Phenotyping in ACS

Acute Coronary Syndromes (ACS) remain a time-critical spectrum in which contemporary pathways, despite high-sensitivity troponins, accelerated emergency department (ED) protocols, and ready access to invasive angiography, still leave clinically meaningful uncertainty at the point of care. The objective is no longer limited to confirming myocardial injury but extends to phenotyping the full ACS cascade: defining the plaque substrate, identifying the culprit mechanism, and quantifying the myocardial consequence that determines prognosis and guides secondary prevention [1,2,3].

It is essential to acknowledge that invasive coronary angiography remains the undisputed gold standard for the evaluation and treatment of patients presenting with ACS and obstructive coronary artery disease. Emergent catheterisation provides an anatomical diagnosis, which can also be aided by the use of intravascular imaging. Moreover, it provides the opportunity for immediate revascularisation, and its central role in the ACS pathway is firmly established by international guidelines [1,2]. However, angiography does not always reveal significant obstructive lesions, as occurs in approximately 5–15% of ACS presentations (MINOCA). Similarly, when the clinician must decide how best to optimise an intervention, plan a complex procedure, or stratify long-term risk, other invasive and non-invasive imaging modalities become indispensable. The “From Plaque to Perfusion” framework is therefore designed not to replace angiography but to address the clinically important questions that angiography alone cannot answer, such as characterising unstable plaque morphology, quantifying myocardial viability, and refining the prognosis of these patients [3,4,5].

This narrative review synthesises high-level evidence from randomized controlled trials and expert consensus documents to establish this clinically pragmatic framework. Rather than viewing imaging modalities in isolation, this approach maps specific technologies to the critical decision points that define the ACS patient journey from emergency department triage and catheterization laboratory intervention to post-event tissue characterisation. Crucially, artificial intelligence (AI) now serves as an essential enabling layer across this continuum. By standardising interpretation and automating complex quantification, AI transforms multimodal imaging outputs into reproducible, actionable metrics that support precision decision-making at scale [6,7,8,9].

## 2. Objectives

The primary objective of this narrative review is to introduce and validate the “From Plaque to Perfusion” framework as a clinically pragmatic model for integrating multimodality imaging in the management of acute coronary syndromes.

The secondary objectives are:

1. To summarise the current evidence base for each imaging modality within this framework, including CCTA, FFR-CT, PET-CT, OCT, IVUS, and CMR.
2. To evaluate the role of artificial intelligence as an enabling technology that enhances diagnostic precision, automates quantification, and supports scalable decision-making across the ACS continuum.
3. To assess the application of multimodality imaging in the specific clinical scenario of Myocardial Infarction with Non-Obstructive Coronary Arteries (MINOCA).
4. To identify current limitations and future research directions, including ongoing trials.

## 3. Methodology

This narrative review is based on a comprehensive literature search conducted across major medical databases, including PubMed/MEDLINE, Scopus, Embase, and Google Scholar, covering the period from January 2010 to February 2026. The search was conducted on 15 December 2025.

Search Strategy: The primary search line combined keywords related to the clinical condition and imaging modalities: (“Acute Coronary Syndrome” OR “Myocardial Infarction” OR “Unstable Angina”) AND (“Coronary Computed Tomography Angiography” OR “CCTA” OR “Optical Coherence Tomography” OR “OCT” OR “Intravascular Ultrasound” OR “IVUS” OR “Cardiac Magnetic Resonance” OR “CMR” OR “Fractional Flow Reserve” OR “FFR-CT” OR “PET-CT”). A secondary search line focused on the role of emerging technologies: (“Artificial Intelligence” OR “Machine Learning” OR “Deep Learning”) AND (“Coronary Imaging” OR “Plaque Characterization” OR “PCI Optimization”). Additional targeted searches were performed for specific trial names (e.g., PLATFORM, ADVANCE, FORECAST, OCTOBER, OCTIVUS, ILUMIEN, PROSPECT, CLIMA, CRISP-CT, ORFAN).

Inclusion Criteria: The review included evidence from the following studies: (a) randomized controlled trials (RCTs) comparing imaging-guided strategies; (b) large prospective natural history and registry studies; (c) systematic reviews and meta-analyses; (d) international expert consensus documents and clinical practice guidelines from the European Society of Cardiology (ESC), the American College of Cardiology (ACC), and affiliated societies; and (e) pivotal studies on AI applications in cardiovascular imaging.

Exclusion Criteria: The following were excluded: (a) single case reports and small case series (fewer than 20 patients); (b) articles not published in English; (c) preclinical or animal studies; (d) editorials, letters, and commentaries without original data; and (e) studies focused exclusively on non-coronary cardiac imaging (e.g., valvular or congenital heart disease).

Reference lists of included articles were hand-searched to identify additional relevant studies. Given the narrative design, no formal risk-of-bias assessment or pooled statistical analysis was performed.

## 4. Results

### 4.1. The “From Plaque to Perfusion” Framework

There are typically three core clinical questions central to the management of acute coronary syndromes. The first question, “What is the plaque substrate?” is addressed by non-invasive coronary CT angiography (CCTA) and PET-CT. These modalities move assessment beyond simple stenosis severity to quantify plaque burden, composition, and high-risk phenotypes. AI-based plaque analytics, exemplified by platforms such as Cleerly, apply FDA-cleared machine-learning algorithms to coronary CT angiography (CCTA) to non-invasively quantify coronary atherosclerosis, stenosis severity, and the likelihood of ischaemia. The platform generates patient-specific 3D coronary models, delineates the lumen and vessel wall, detects and measures stenoses, and quantifies plaque; its ischemia assessment is derived from models trained against invasive fractional flow reserve (FFR) data to estimate vessel-level ischemia risk [8,9]. Furthermore, CT-FFR algorithms provide non-invasive fractional flow reserve derived from coronary CT angiography, adding lesion-specific functional assessment to anatomical CCTA in suspected ACS/acute chest pain pathways. This can help identify flow-limiting disease, guide triage to invasive angiography versus conservative management, and support PCI planning by localising physiologically significant stenoses.

The second question, “What is the culprit mechanism?” is resolved in the catheterization laboratory using intravascular imaging. Optical coherence tomography (OCT) and intravascular ultrasound (IVUS) distinguish atherosclerotic from non-atherosclerotic causes (e.g., spontaneous coronary artery dissection) and refine mechanistic diagnosis (rupture, erosion, calcified nodule). IVUS uses ultrasound, giving deeper tissue penetration and comprehensive assessment of overall plaque burden and vessel remodeling (with lower spatial resolution). OCT uses near-infrared light, offering much higher resolution for detailed luminal and superficial plaque features (e.g., cap thickness, thrombus, stent apposition) but with shallower penetration and the need for blood clearance. Recent advances in AI-enhanced systems now automate lumen and vessel segmentation, reducing analysis time and standardizing PCI optimisation endpoints [10,11,12,13].

**Figure 1:**
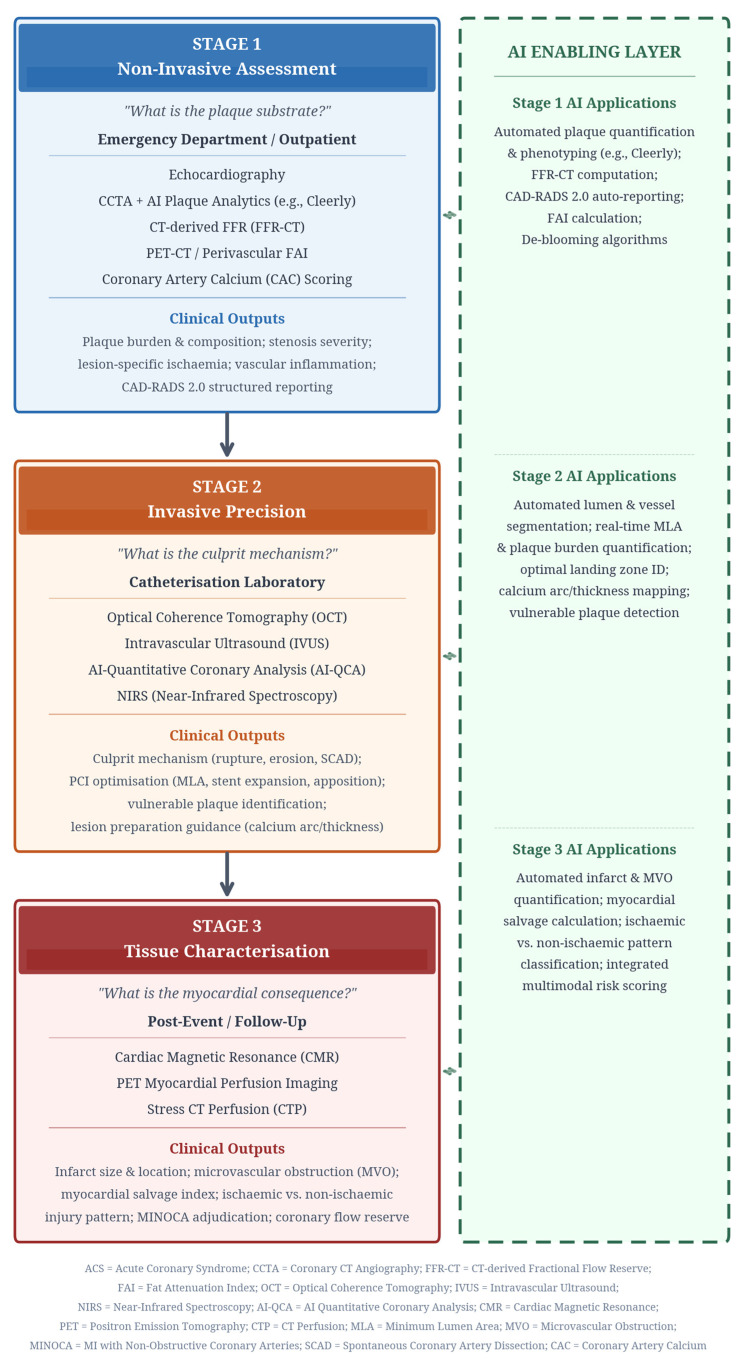
The “From Plaque to Perfusion” Framework. The framework maps multimodality imaging to three sequential clinical questions in acute coronary syndrome management. Stage 1 (non-invasive assessment) defines the plaque substrate using CCTA, FFR-CT, and PET-CT. Stage 2 (invasive precision) resolves the culprit mechanism via OCT and IVUS in the catheterisation laboratory. Stage 3 (tissue characterisation) quantifies the myocardial consequence using CMR and PET. Artificial intelligence serves as an enabling layer across all stages, automating analysis and standardising interpretation.

The third question, “What is the myocardial consequence?” is anchored by cardiac magnetic resonance (CMR). CMR quantifies infarct size, microvascular injury, and myocardial salvage, which are critical for risk stratification and adjudicating complex entities such as MINOCA (Myocardial Infarction with Non-Obstructive Coronary Arteries) [14,15]. Emerging data for CT with myocardial perfusion (stress CTP, often alongside CCTA ± CT-FFR) can identify flow-limiting CAD by showing stress-induced perfusion defects while simultaneously defining coronary anatomy, enabling a single-session “anatomy + ischemia” assessment [16]. The overall structure of this framework is illustrated in Figure 1.

### 4.2. Summary of Key Evidence

Table 1 summarises the principal studies and meta-analyses identified in this review, organised by research direction.

### 4.3. Advanced Non-Invasive Assessment: AI-Powered Coronary CT Angiography

CCTA is evolving from a “rule-out” test into a comprehensive non-invasive phenotyping platform for ACS evaluation. In acute chest pain pathways, basic modalities like echocardiography remain essential for assessment of wall motion abnormalities. CCTA builds on this by increasing diagnostic certainty and facilitating safe, accelerated triage when integrated with clinical assessment, ECG, and biomarkers [17,18,19,35]. Coronary artery calcium (CAC) scoring provides a measure of cumulative plaque burden, while CT-derived fractional flow reserve (FFR-CT) adds lesion-specific physiology. AI augments these by standardizing interpretation and enabling reproducible quantification of coronary anatomy and plaque features, supporting structured reporting such as CAD-RADS 2.0 and reducing inter-observer variability [4,35]. Evidence from the PLATFORM, ADVANCE, and FORECAST trials supports the clinical utility of FFR-CT in reducing unnecessary invasive angiography [20,21,22]. A detailed comparison of these three trials is presented in Table 2.

The evolution of precision phenotyping is perhaps best illustrated by the “P-series” of trials. In the PROSPECT II (P2) study, comprehensive three-vessel NIRS–IVUS imaging following myocardial infarction demonstrated that a significant proportion of subsequent adverse events originate from angiographically non-obstructive, untreated non-culprit lesions. Specifically, a large plaque burden (detected by IVUS) and a lipid-rich core (detected by NIRS) were identified as key predictors of future major adverse cardiovascular events (MACE). The study reported a 4-year non-culprit MACE rate of 13.2% in patients with at least one high-risk lesion, compared to 7.0% for lesions characterized by both large plaque burden and a substantial lipid core [24].

Building on these insights, the focus is now shifting toward pre-procedural planning using CCTA. The Precise Procedural and PCI Plan (P4) trial is currently comparing CCTA-guided strategies against traditional IVUS-guided PCI with longitudinal follow-up. Similarly, the OPTIMAL (P5) programme extends this CT-guided planning to the challenging subset of severely calcified coronary artery disease; definitive comparative clinical outcomes are still awaited.

CT and PET-CT enable phenotyping of plaque biology and vascular inflammation. A key CT-derived biomarker is the perivascular fat attenuation index (FAI), which captures inflammation-driven compositional changes in pericoronary adipose tissue and provides a non-invasive readout of coronary inflammatory activity. In the original CRISP-CT programme, higher FAI was associated with increased cardiac mortality independent of traditional risk factors, supporting incremental prognostic value beyond anatomy alone. More recently, the large multicentre ORFAN longitudinal cohort extended these findings to patients undergoing clinically indicated CCTA, showing that most events occurred in individuals without obstructive CAD, and that an elevated FAI Score across the major coronary arteries substantially strengthened prediction of cardiac mortality and MACE [27,28].

Key challenges in CCTA include motion artifacts and “blooming” from heavy calcification. These are increasingly mitigated through high-temporal resolution scanners, AI-based de-blooming algorithms, and the use of FFR-CT to adjudicate the functional significance of calcified lesions [16]. 

### 4.4. The Shift Beyond Angiography: Intelligent Intravascular Imaging

Intravascular imaging provides lesion-level phenotyping that angiography cannot reliably deliver, supporting precision PCI.

While both modalities improve outcomes, they offer complementary strengths. OCT provides superior axial resolution (10–20 µm), making it ideal for identifying plaque rupture, erosion, and stent-related pathology. IVUS offers deeper penetration, which is advantageous for vessel sizing in large vessels or left main disease. The OCTIVUS trial recently demonstrated that OCT-guided PCI is non-inferior to IVUS-guided PCI regarding target-vessel failure [10,11,12].

The ILUMIEN III: OPTIMIZE PCI trial showed that OCT guidance achieves similar stent expansion to IVUS, while the ULTIMATE trial confirmed the superiority of IVUS-guided PCI over angiography alone in improving clinical outcomes. More recently, the OCTOBER trial highlighted the benefits of OCT guidance in complex bifurcation lesions [11,29,30]. In patients with severe renal impairment, Japanese studies have emphasized that ultra-low-contrast or zero-contrast PCI protocols using OCT with low-molecular-weight dextran or saline flushes can minimize the risk of contrast-induced acute kidney injury [36,37].

Furthermore, in patients with prior coronary artery bypass grafting (CABG), imaging is critical for distinguishing degenerative graft disease from anastomotic stenosis and guiding embolic protection strategies [30]. A major shift is the move toward pre-procedural planning using CCTA. The P3 study and the ongoing P4 and P5 trials are evaluating whether CT-guided PCI strategies can match or exceed the precision of intravascular imaging-guided approaches [33,34]. Additionally, the BYPASS-CTCA trial was a randomised study in patients with previous CABG undergoing invasive angiography, testing whether performing CT coronary angiography first improves the subsequent catheterisation procedure. It showed that a CT-guided strategy made angiography faster, safer, and more efficient, with lower contrast use and radiation exposure, supporting CCTA as a useful planning tool in post-CABG patients.

The integration of AI into intravascular imaging platforms represents a paradigm shift in the catheterization laboratory. Beyond simple visualization, AI-driven algorithms now provide real-time, automated lumen and vessel quantification, which is instrumental in high-stakes clinical decision-making. By accurately determining the minimum lumen area and plaque burden, AI assists clinicians in adjudicating between medical management and intervention, as well as choosing between PCI and CABG in complex multivessel disease. Furthermore, imaging enhances procedural precision by identifying optimal “landing zones” in challenging scenarios such as ostial lesions, where precise stent placement is critical. It also guides lesion preparation techniques—such as the need for rotational atherectomy or intravascular lithotripsy—by quantifying calcium arc and thickness, thereby ensuring optimal stent expansion and reducing the risk of procedural complications [10,11,12,13].

Intracoronary imaging can identify non-culprit plaques with high-risk morphology that predict future coronary events, as shown in natural-history studies such as PROSPECT (VH-IVUS) and PROSPECT II (NIRS-IVUS, where large plaque burden plus high lipid signal enriched for later events) and in OCT cohorts such as CLIMA (where clustered OCT features like thin cap, large lipid arc, macrophages, and small MLA were associated with subsequent events). Building on this, AI-enabled OCT/IVUS pipelines can automate frame- and pullback-level detection/quantification of high-risk phenotypes (e.g., TCFA and adverse plaque composition) across long vessel segments—including non-culprit territories—making systematic “whole-vessel” risk phenotyping feasible and less operator-dependent. Clinically, the most immediate value is risk enrichment to target more intensive preventive therapy (lipid lowering, risk-factor optimisation, adherence), while plaque-directed prophylactic intervention remains investigational (e.g., PROSPECT ABSORB), but conceptually could reduce future events if outcomes trials confirm benefit [23,24,25,26].

### 4.5. Special Clinical Scenario: MINOCA

Myocardial Infarction with Non-Obstructive Coronary Arteries (MINOCA) is a heterogeneous clinical entity that necessitates a systematic, multimodality imaging approach to transition from a “working diagnosis” to a definitive mechanistic cause.

CMR is the gold standard for tissue-level adjudication in MINOCA, ideally performed within 7–14 days of the index event. Its ability to differentiate between ischaemic and non-ischaemic patterns of myocardial injury is paramount. A subendocardial or transmural pattern of late gadolinium enhancement (LGE) confirms an ischaemic mechanism (e.g., plaque disruption or thromboembolism), whereas subepicardial or mid-wall LGE typically points toward non-ischaemic causes such as myocarditis. Furthermore, CMR can identify Takotsubo syndrome through characteristic wall motion abnormalities and the absence of significant LGE, as well as quantify microvascular injury and myocardial salvage [14,15].

When CMR suggests an ischaemic origin but angiography remains inconclusive, intravascular imaging (OCT or IVUS) becomes essential to identify subtle epicardial substrates. OCT, with its near-histologic resolution, is particularly adept at detecting “angiographically silent” plaque disruptions, erosions, or spontaneous coronary artery dissection (SCAD). Identifying these substrates is critical, as it shifts management from generic secondary prevention to targeted antithrombotic or mechanical strategies. The MINOCA-BAT and PROSPECT studies have underscored that a significant proportion of MINOCA cases involve underlying plaque events that are only visible through endovascular phenotyping [15,23,31].

Beyond the acute phase, PET-CT and advanced CCTA biomarkers offer insights into coronary inflammatory activity and microvascular function. PET myocardial perfusion imaging (MPI) provides high diagnostic accuracy for assessing microvascular ischemia in patients with no obstructive coronary arteries (INOCA), a population that frequently overlaps with MINOCA. Additionally, CCTA-derived perivascular fat attenuation index (FAI) can identify localized vascular inflammation, potentially flagging “vulnerable” patients who remain at risk for recurrent events despite the absence of flow-limiting stenosis [27,28,32].

### 4.6. Aligning the Framework with Clinical Practice Guidelines

The “From Plaque to Perfusion” framework is designed to be directly actionable by mapping its components to established Class I and Class IIa recommendations from the European Society of Cardiology (ESC) and the American College of Cardiology (ACC). This alignment ensures that the proposed imaging pathway complements, rather than contradicts, current standards of care. Table 3 summarises the current guideline recommendations supporting the use of imaging at each stage of the framework.

## 5. Discussion

### 5.1. Comparative Analysis of Non-Invasive Assessment Strategies

The evidence base for CCTA in ACS has evolved substantially over the past decade. Early trials such as ROMICAT-II, CT-ACS, and CT-STAT established CCTA as a safe and efficient “rule-out” tool in the emergency department, demonstrating reduced time to diagnosis and shorter hospital stays without an increase in adverse events [17,18,19]. However, these studies primarily evaluated CCTA’s ability to exclude obstructive disease rather than to characterize plaque biology or guide downstream management.

The subsequent generation of FFR-CT trials—PLATFORM, ADVANCE, and FORECAST—marked a paradigm shift by adding lesion-specific physiology to anatomical assessment. PLATFORM demonstrated that an FFR-CT-guided strategy significantly reduced the rate of invasive coronary angiography showing no obstructive disease, translating into cost savings at one year [20]. ADVANCE extended these findings into real-world practice, confirming that abnormal FFR-CT values were associated with higher rates of revascularization and, importantly, with higher rates of cardiovascular death and myocardial infarction, thereby validating its prognostic utility [21]. FORECAST, conducted within the UK National Health Service, showed that while overall cost savings were not statistically significant, the FFR-CT strategy substantially reduced the need for invasive angiography [20]. Taken together, these three trials demonstrate a consistent trajectory: CCTA has evolved from a binary gatekeeper into a comprehensive, physiology-informed decision tool.

The integration of inflammatory biomarkers further extends the non-invasive phenotyping capability of CCTA. The CRISP-CT study was the first to demonstrate that perivascular FAI independently predicts cardiac mortality, while the larger ORFAN cohort confirmed that most cardiovascular events occur in patients without obstructive disease—a population in which FAI provides critical incremental prognostic value [27,28]. These findings have profound implications for risk stratification, particularly in MINOCA and INOCA populations where traditional anatomical assessment is insufficient.

### 5.2. Comparative Analysis of Intravascular Imaging Strategies

The landscape of intravascular imaging has been transformed by a series of landmark trials. The ILUMIEN III trial in 2016 first demonstrated that OCT-guided stent implantation achieves comparable stent expansion to IVUS, with both modalities outperforming angiography alone [29]. This was followed by the ULTIMATE trial, which confirmed the clinical superiority of IVUS-guided PCI over angiography-guided PCI, establishing intravascular imaging as a standard of care rather than an optional adjunct [30].

More recently, three pivotal trials published in 2023 have refined the comparative landscape. The ILUMIEN IV trial showed that OCT guidance improved minimum stent area compared to angiography, although the primary clinical endpoint of target-vessel failure did not reach statistical significance at two years [10]. The OCTOBER trial provided compelling evidence for OCT guidance in complex bifurcation lesions, a scenario where precise visualization of side-branch compromise and stent positioning is critical [11]. Perhaps most importantly, the OCTIVUS trial directly compared OCT-guided and IVUS-guided PCI for the first time in a randomized setting, demonstrating non-inferiority of OCT to IVUS for one-year target-vessel failure [12]. The 2026 network meta-analysis by Carvalho and colleagues synthesised this body of evidence, confirming that both OCT and IVUS are superior to angiography guidance and broadly comparable to each other, while acknowledging that each modality retains specific clinical niches—OCT for superficial plaque characterization and stent optimization, IVUS for deep tissue assessment and left main disease [13].

### 5.3. From Vulnerable Plaque to Guided Intervention:

The natural history studies provide the scientific rationale for the emerging concept of plaque-directed therapy. The original PROSPECT study (2011) was the first large-scale prospective study to demonstrate that VH-IVUS could identify non-culprit plaques destined to cause future events [23]. PROSPECT II extended this work by combining NIRS with IVUS, showing that the combination of large plaque burden and high lipid content substantially enriched for future non-culprit MACE [24]. In parallel, the CLIMA study demonstrated that OCT-derived features—thin fibrous cap, large lipid arc, macrophage infiltration, and small minimum lumen area—were independently associated with adverse outcomes [25]. These complementary findings from IVUS-based and OCT-based studies converge on a common conclusion: high-risk plaque phenotypes can be reliably identified and may serve as targets for intensified preventive therapy. The PROSPECT ABSORB trial took the next step by evaluating prophylactic treatment of vulnerable plaques with bioresorbable scaffolds, though this approach remains investigational pending larger outcomes trials [26].

### 5.4. Multimodality Imaging in MINOCA: An Integrated Diagnostic Algorithm

The MINOCA population exemplifies the necessity of the “From Plaque to Perfusion” framework. Angiography, by definition, fails to identify the underlying cause in these patients. The evidence reviewed demonstrates that CMR provides tissue-level diagnosis in the majority of MINOCA cases, differentiating ischaemic from non-ischaemic injury patterns [14,15]. When CMR suggests an ischaemic mechanism, intravascular imaging—particularly OCT—can identify subtle epicardial substrates such as plaque erosion or SCAD that are invisible on angiography. Reynolds and colleagues demonstrated that the combined use of OCT and CMR identified a definitive underlying cause in the majority of women presenting with MINOCA, underscoring the value of a systematic, multimodality approach [31]. For patients with suspected microvascular dysfunction, PET-CT provides quantitative assessment of coronary flow reserve, with Taqueti and colleagues demonstrating that PET-detected microvascular dysfunction independently predicts future heart failure hospitalization [32].

## 6. Strengths, Limitations, and Future Directions

### 6.1. Strengths

The principal strength of the “From Plaque to Perfusion” framework is its clinical pragmatism. Rather than presenting imaging modalities as isolated diagnostic tools, it maps imaging choices to specific management decisions across the ACS pathway, integrating non-invasive plaque and physiologic assessment, catheterization laboratory mechanistic diagnosis, and post-event tissue phenotyping. The framework is grounded in high-level evidence from recent RCTs and international guidelines, and it explicitly incorporates the role of AI as an enabling technology that makes this integrated approach scalable and reproducible.

### 6.2. Limitations

This is a narrative review rather than a systematic review; therefore, it does not provide pooled effect estimates or formal risk-of-bias assessment. The selection of studies, while comprehensive, may be subject to selection bias inherent to the narrative review methodology. Additionally, access to advanced modalities such as FFR-CT, PET-CT, and AI-enhanced platforms varies substantially across health systems, and the generalizability of the framework may be limited in resource-constrained settings. AI tools, while promising, require local validation, regulatory approval, and governance frameworks before widespread clinical adoption.

### 6.3. Future Directions

Future work should prioritize prospective trials linking imaging-derived plaque and inflammation metrics to therapy escalation and clinical outcomes. The ongoing P4 and P5 CT-guided PCI programmes represent the next frontier in “wire-free” procedural planning and may fundamentally alter how interventional procedures are designed. Automated intravascular analysis using AI is likely to expand, with increasing emphasis on standardized, quantitative endpoints that can be audited and improved. Additionally, the integration of multimodal data streams—combining anatomical, physiological, and inflammatory biomarkers into unified risk scores—represents a promising avenue for truly personalized ACS management.

## 7. Conclusions

This narrative review introduces and validates the “From Plaque to Perfusion” framework as an integrated, mechanism-driven pathway for multimodality imaging in acute coronary syndromes. The following specific conclusions are drawn in relation to the stated objectives:

First, regarding non-invasive assessment: CCTA, augmented by AI-driven plaque analytics and FFR-CT, has evolved from a simple rule-out tool into a comprehensive phenotyping platform. The PLATFORM, ADVANCE, and FORECAST trials collectively demonstrate that FFR-CT reduces unnecessary invasive angiography, while the CRISP-CT and ORFAN studies confirm that CT-derived inflammatory biomarkers provide incremental prognostic value beyond anatomy alone.

Second, regarding invasive procedural guidance: Intravascular imaging with OCT and IVUS is superior to angiography alone for optimizing PCI, as confirmed by the ILUMIEN III, ILUMIEN IV, ULTIMATE, OCTOBER, and OCTIVUS trials, and synthesised in the 2026 network meta-analysis. AI-enhanced platforms further augment procedural precision by automating lumen quantification, identifying optimal landing zones, and guiding lesion preparation strategies.

Third, regarding post-event tissue characterization: CMR remains the gold standard for quantifying myocardial injury and differentiating ischaemic from non-ischaemic causes in MINOCA. When combined with intravascular imaging and PET-CT, a systematic multimodality approach can identify a definitive mechanistic cause in the majority of MINOCA patients, enabling targeted rather than empirical secondary prevention.

Fourth, regarding the role of AI: Artificial intelligence serves as the essential enabling layer that makes this integrated framework scalable. By standardising interpretation, automating quantification, and integrating multimodal outputs into reproducible metrics, AI transforms ACS imaging from a series of discrete diagnostic tests into a unified decision pathway that supports precision, phenotype-led cardiovascular care.

## Figures and Tables

**Table 1:** Summary of Key Clinical Trials and Meta-Analyses Supporting the “From Plaque to Perfusion” Framework.

Research Direction	Key Study/Trial	Main Author	Year	Primary Objective	Key Result/Finding
CCTA in acute chest pain	ROMICAT-II	Hoffmann U	2012	Compare CCTA vs. standard evaluation in ED	CCTA reduced time to diagnosis and length of stay without increasing downstream costs [17]
CCTA in acute chest pain	CT-ACS	Litt HI	2012	Assess CCTA for safe discharge of ACS patients	CCTA safely identified low-risk patients for early discharge [18]
CCTA in acute chest pain	CT-STAT	Goldstein JA	2011	Evaluate CCTA vs. MPI for acute chest pain	CCTA reduced diagnostic time and was cost-effective compared to MPI [19]
FFR-CT clinical utility	PLATFORM	Douglas PS	2016	Assess if FFR-CT reduces unnecessary ICA	FFR-CT strategy reduced unnecessary ICA and lowered costs at 1 year [20]
FFR-CT clinical utility	ADVANCE	Patel MR	2020	Relate FFR-CT to downstream management	Revascularization more frequent when FFR-CT ≤ 0.80; low overall MACE [21]
FFR-CT clinical utility	FORECAST	Curzen N	2021	Compare CCTA + FFR-CT vs. standard care (UK)	ICA reduced in CCTA + FFR-CT arm; QoL and major events similar [22]
Vulnerable plaque identification	PROSPECT	Stone GW	2011	Natural history of coronary atherosclerosis	VH-IVUS identified high-risk non-culprit plaques predicting future events [23]
Vulnerable plaque identification	PROSPECT II	Erlinge D	2021	Identify predictors of future non-culprit MACE	High plaque burden (IVUS) + large lipid core (NIRS) enriched for future MACE [24]
Vulnerable plaque identification	CLIMA	Prati F	2020	Assess OCT plaque features and outcomes	Clustered OCT features (thin cap, large lipid arc, macrophages, small MLA) predicted events [25]
Prophylactic plaque intervention	PROSPECT ABSORB	Stone GW	2020	Evaluate bioresorbable scaffold for vulnerable plaques	Demonstrated feasibility; plaque-directed intervention remains investigational [26]
Coronary inflammation	CRISP-CT	Oikonomou EK	2018	Assess if perivascular FAI predicts cardiac mortality	High FAI independently associated with increased cardiac mortality [27]
Coronary inflammation	ORFAN	Chan K	2024	Assess FAI in patients without obstructive CAD	Elevated FAI Score strengthened prediction of cardiac mortality and MACE [28]
OCT-guided PCI	ILUMIEN III	Ali ZA	2016	Compare OCT vs. IVUS vs. angiography for stent implantation	OCT achieved similar stent expansion to IVUS; both superior to angiography [29]
OCT-guided PCI	ILUMIEN IV	Ali ZA	2023	Compare OCT-guided vs. angiography-guided PCI	OCT guidance improved minimum stent area but did not reduce 2-year TVF [10]
OCT-guided PCI	OCTOBER	Holm NR	2023	Evaluate OCT guidance in complex bifurcation PCI	OCT guidance resulted in superior procedural outcomes [11]
OCT vs. IVUS guidance	OCTIVUS	Kang DY	2023	Compare OCT vs. IVUS guidance for PCI	OCT was non-inferior to IVUS for 1-year target-vessel failure [12]
IVUS-guided PCI	ULTIMATE	Zhang J	2018	Compare IVUS-guided vs. angiography-guided DES implantation	IVUS guidance superior to angiography alone for clinical outcomes [30]
Imaging-guided PCI (network)	Network meta-analysis	Carvalho PEP	2026	Compare IVUS, OCT, and angiography guidance	Both IVUS and OCT superior to angiography; comparable to each other [13]
CMR in MINOCA	Sörensson P	Sörensson P	2021	Early CMR in MINOCA patients	CMR identified a definitive diagnosis in the majority of MINOCA cases [14]
MINOCA outcomes	Meta-analysis	Pustjens T	2020	Systematic review of MINOCA outcomes	MINOCA carries significant morbidity; multimodality imaging improves diagnosis [15]
MINOCA in women	Reynolds HR	Reynolds HR	2021	OCT + CMR to determine MINOCA causes in women	Combined OCT and CMR identified the underlying cause in most women [31]
PET in INOCA	Taqueti VR	Taqueti VR	2018	Assess PET for microvascular dysfunction	PET-detected microvascular dysfunction predicted heart failure hospitalisation [32]
CT-guided PCI planning	P3 study design	Sonck J	2021	Design of CT-guided PCI planning study	Established rationale for CCTA-based procedural planning [33]
CT-guided PCI planning	P4 trial	ClinicalTrials.gov	2022	Compare CCTA-guided vs. IVUS-guided PCI	Ongoing; definitive outcomes awaited [34]

**Table 2:** Comparative Summary of Landmark FFR-CT Trials: PLATFORM, ADVANCE, and FORECAST.

	Platform	Advance	Forecast
Clinical question	Prospective, multicenter comparative-effectiveness study (patients managed by usual care vs. CCTA with selective FFR-CT; includes a “planned invasive” stratum)	In real-world practice, how does FFR-CT (≤0.80 vs. >0.80) relate to downstream management (revascularisation) and outcomes after CCTA shows atherosclerosis?	In UK rapid access chest pain clinics, does CCTA + selective FFR-CT improve resource utilization/costs vs. NICE-guided standard care?
Primary endpoint	At 90 days: rate of ICA showing no obstructive CAD (i.e., “unnecessary” cath)	Relationship of FFR-CT to downstream care + 1-year clinical outcomes (MACE, death/MI, etc.)	Total cardiac costs at 9 months
Results	In the planned invasive stratum, lower costs at 1 year with CCTA + selective FFR-CT and similarly low MACE/QoL vs. usual care	Revascularization much more frequent when FFR-CT ≤ 0.80; event rates are overall low, with higher CV death/MI in abnormal vs. normal FFR-CT	No significant cost reduction, but ICA was reduced in the CCTA + selective FFR-CT arm; QoL/angina and major events were similar

**Table 3:** Current guidelines recommendations to support the use of imaging in CAD.

Guideline Recommendation	Class	Framework Stage	Clinical Application
CCTA for ACS Rule-Out	I (ESC/ACC)	Stage 1: Non-Invasive Assessment	In low-to-intermediate risk patients with suspected ACS and no ischaemic ECG changes or elevated troponins, CCTA is recommended to rule out obstructive CAD [1,2,7].
Intravascular Imaging for PCI Optimization	IIa (ESC)	Stage 2: Invasive Precision	Intravascular imaging (IVUS or OCT) should be considered to optimize stent implantation, assess stent expansion, malapposition, and edge dissections, particularly in complex lesions [1,38].
CMR for MINOCA Evaluation	I (ESC)	Stage 3: Tissue Characterization	In patients with MINOCA, CMR is recommended to establish the underlying cause by differentiating ischaemic from non-ischaemic myocardial injury [1,3].
FFR-CT for Functional Assessment	IIa (ESC)	Stage 1: Non-Invasive Assessment	FFR-CT may be considered in patients with intermediate-stenosis lesions on CCTA to determine haemodynamic significance and guide decisions regarding invasive angiography [7].

## Data Availability

The original contributions presented in this study are included in the article. Further inquiries can be directed to the corresponding authors.
